# Chemotherapy-induced toxic epidermal necrolysis in a patient with multiple myeloma, a case report and literature review

**DOI:** 10.3389/fonc.2023.1227448

**Published:** 2023-08-02

**Authors:** Rui X, Meidan W, Gongqiang W, Longyi Z, Xiaoxia W, Wei C, Chenhui W

**Affiliations:** ^1^ Department of Hematology, Dongyang Hospital Affiliated with Wenzhou Medical University, Dongyang, Zhejiang, China; ^2^ Faculty of Biology, University of Freiburg, Freiburg, Germany; ^3^ Clinical Laboratory, Dongyang Hospital Affiliated to Wenzhou Medical University, Dongyang, Zhejiang, China

**Keywords:** toxic epidermal necrolysis, Stevens–Johnson syndrome, lenalidomide, adverse drug reaction, multiple myeloma, tumor microenvironment

## Abstract

**Rationale and patient concerns:**

Toxic epidermal necrolysis (TEN) and Stevens-Johnson syndrome (SJS) are severe drug-induced skin reactions associated with a high mortality rate. The patient in this case report developed TEN after receiving the Velcade-lenalidomide-dexamethasone (VRD) regimen for the treatment of multiple myeloma (MM). The patient’s concerns included the progression of the rash, pain, itching, and potential long-term complications. TEN is a life-threatening condition that requires prompt medical intervention and hospitalization.

**Interventions:**

The treatment approach for the patient included discontinuation of the causative medication (lenalidomide) and comprehensive supportive therapy. Supportive measures included the administration of systemic corticosteroids (methylprednisolone), intravenous immunoglobulin infusion, pain relief medication (ebastine), antibiotic prophylaxis, laminar bed use, and regular dressing changes. The goal was to alleviate symptoms, promote skin and mucous membrane healing, and prevent complications such as infection.

**Diagnosis:**

The patient was diagnosed with stage III A DS and stage III ISS MM, specifically of the immunoglobulin G (λ) type. Diagnostic procedures included CT and MRI scans, bone marrow testing through flow cytometry and morphology analysis, and laboratory tests to assess blood markers. The diagnosis of TEN was made based on the clinical presentation, skin biopsy, and exclusion of other potential causes.

**Outcomes:**

With the implemented interventions, the patient’s condition gradually improved, and the rash resolved without any residual scarring. The patient’s skin and mucosa healed, blood markers improved, and bone pain was relieved. The patient was discharged within a month of receiving the final treatment with bortezomib and dexamethasone. The patient got partial response(PR) of multiple myeloma.

**Lessons:**

Drug-induced SJS/TEN is more prevalent in Asian populations, potentially due to differences in human leukocyte antigen (HLA) alleles. The use of systemic corticosteroid therapy in SJS/TEN cases is controversial due to the potential risks of immune suppression and complications. Balancing the immune response to prevent SJS/TEN while maintaining an effective cytotoxic immune response for tumor control remains a challenge. Lenalidomide, an immunomodulatory agent, can enhance antitumor immune responses but also contribute to the pathogenesis of SJS/TEN. Increased awareness of HLA variations and frequently mutated genes in different malignancies can help prevent SJS/TEN and improve patient outcomes.

## 1 Introduction

Extensive research has been conducted on SJS since its first discovery in the 20th century, and its mechanism of action has been explored over the years. In the clinic, patients always suffer fever, fatigue, and upper respiratory tract symptoms followed by spreading erythema, and even blisters. SJS is classified based on several symptoms, with skin biopsy being the primary diagnostic tool . Similarly, Toxic Epidermal Necrolysis (TEN), more than 30% of the skin surface involved in erosion of mucosae, occurs in a variety of diseases and shares similar symptoms with SJS (1–5). Both conditions represent severe drug-induced skin reactions that can be mediated by the immune response and have a high mortality rate observed in affected patients (6, 7).

Despite the differences in the extent of skin exfoliation between SJS and TEN, they are considered two manifestations of allergic skin reactions. The mortality rate of TEN ranges from 15% to 30%, while SJS has a lower mortality rate (8). Advanced age, comorbidities, sepsis, and hematologic malignancies are associated with higher mortality risk. While skin infections rarely cause death, a retrospective study found that women and patients with prior health complications are more susceptible to SJS, SJS/TEN, and TEN, with women accounting for 62% of SJS/TEN cases (9).

**Figure 1:**
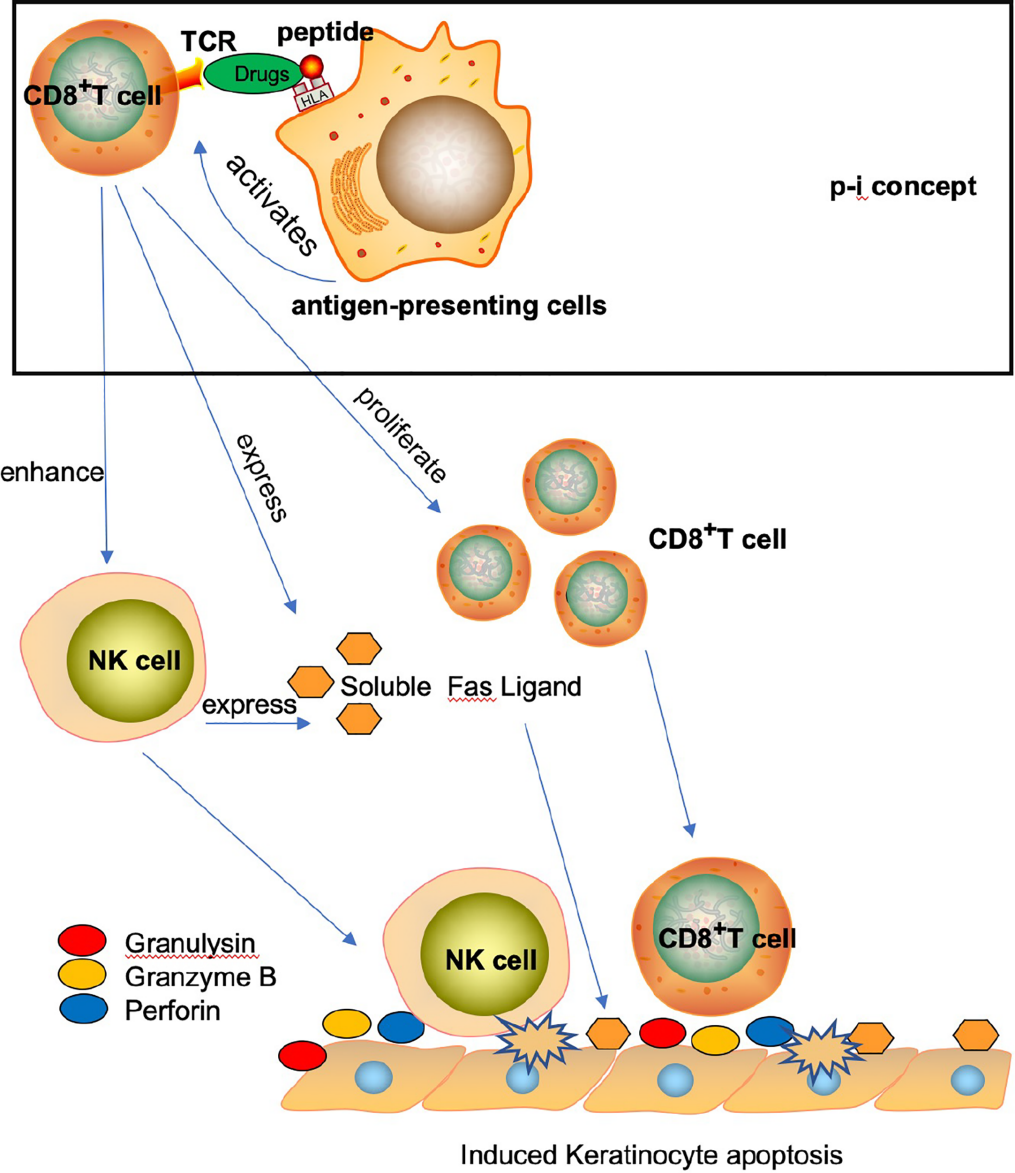
Induced keratinocyte apoptosis.

Over the past century, the mechanisms underlying the mortalities resulting from SJS, SJS/TEN, and TEN have been extensively studied (10, 11). Previous reports have primarily implicated T cells and NK cells in the pathogenesis of these conditions (11, 12). For example, the formation of a drug-HLA molecule-T cell complex (known as the p-i concept) is believed to play a role in the pathogenesis of SJS/TEN, promoting the lysis of keratinocytes through the adaptive immune response, ultimately resulting in the patient’s mortality (13). Additionally, CD8+ T cells and NK cells may promote SJS/TEN through the direct and indirect release of soluble factors such as FAS ligand, perforin, tumor necrosis factors, and granulysin (13–15) (**Figure 1**). The pathogenesis and causes of SJS/TEN vary depending on the drug structure, its pharmacokinetic characteristics, and the diverse adaptive immune responses of patients. While certain drugs can induce the response, activated NK cells may also promote keratinocyte lysis (14).

Patients diagnosed with SJS, SJS/TEN, and TEN require medical treatment and hospitalization. Currently, acetaminophen, opioids, nonsteroidal anti-inflammatory drugs (NSAIDs), and topical anesthetics are widely used to relieve pain and reduce inflammation (16, 17). For example, NSAIDs such as ibuprofen or aspirin can reduce inflammation levels and alleviate the symptoms of these conditions. However, patients with kidney diseases or a history of gastrointestinal ulcers may not benefit from NSAIDs (18, 19). Therefore, there is a strong need for further exploration of how to combine immune with chemotherapy to improve therapeutic outcomes and inhibit SJS, SJS/TEN, and TEN occurrences.

## 2 Case report

A 69-year-old male patient with a medical history of type II diabetes and hypertension was diagnosed with stage III A DS and stage III ISS MM, specifically of the immunoglobulin G (λ) type. The patient presented with chest pain attributed to an osteolytic lesion affecting the ribs and thoracic vertebrae. The patient underwent treatment with the VRD regimen, consisting of daily administration of lenalidomide (25 mg) from day 1 to 21, bortezomib (2.1 mg) on days 1, 4, 8, and 11, and dexamethasone (20 mg) on days 1, 2, 4, 5, 8, 9, 11, and 12, aiming to improve therapeutic outcomes.

Diagnostic procedures included CT and MRI scans, as well as bone marrow testing through flow cytometry and morphology analysis. The MRI scans revealed abnormal signals indicating multiple bone destruction in the thoracic spine and ribs, distinguishing the patient from extramedullary plasma cell tumor lesions. The bone marrow morphology analysis indicated the presence of 36% abnormal plasma cells, and flow cytometry detected 11.3% monoclonal plasma cells expressing CD38, CD138, and lambda restriction. The patient’s karyotype was determined to be normal (46, XY). Fluorescence *in situ* hybridization (FISH) analysis revealed an IGH translocation in 40% of the cells. Immunofixation detected an electric IgG lambda positivity. Additional laboratory findings included a serum IgG level of 65.3 g/L, IgA level below 0.25 g/L, IgM level below 0.16 g/L, HGB level of 73 g/L, LDH level of 248 U/L, β-2 microglobulin level of 7.8 mg/L, and other abnormal blood and urea test results. The MRI scans also identified more than three osteolytic lesions affecting the thoracic and lumbar vertebrae, as well as the ribs.

**Figure 2:**
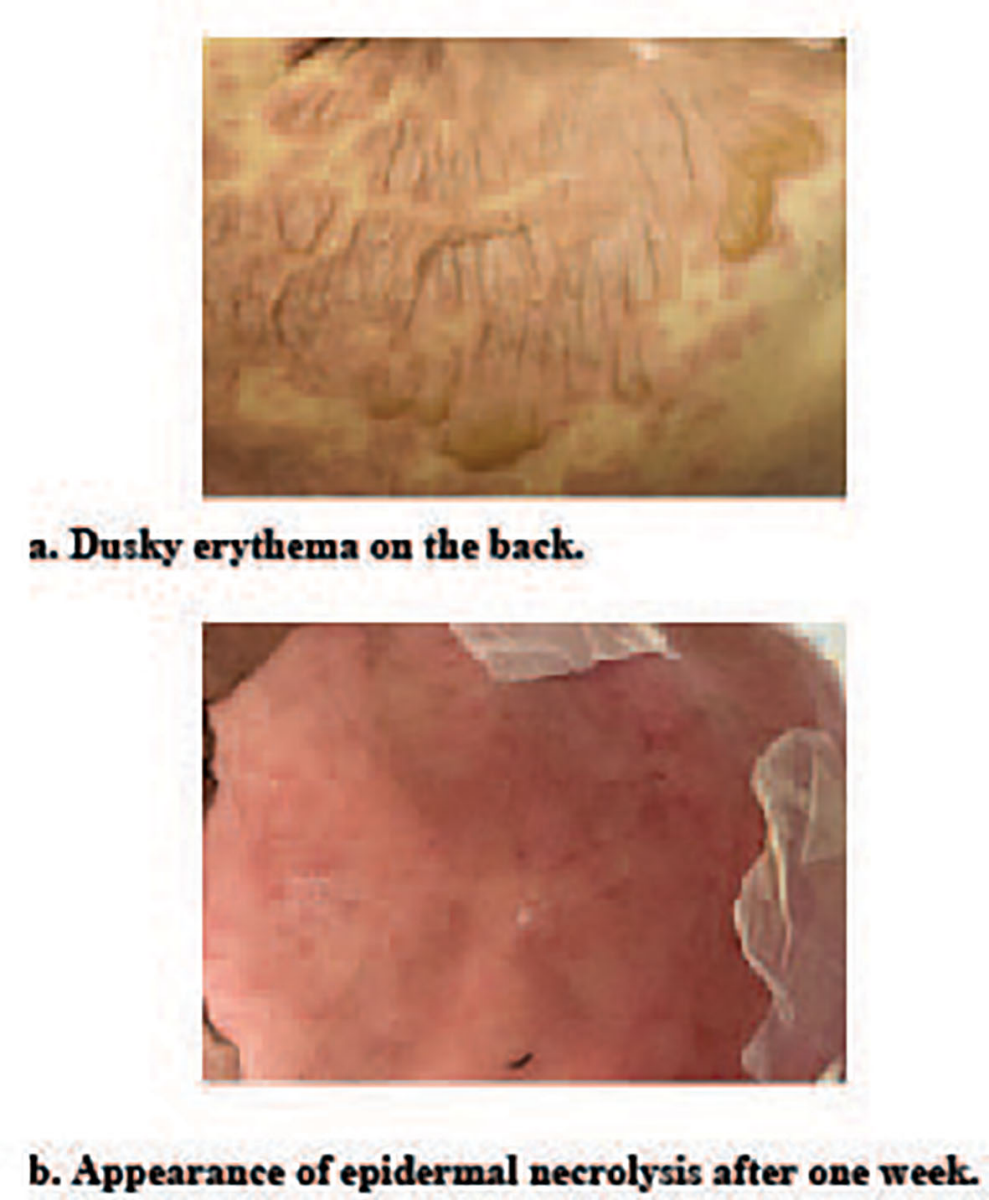
Toxic epidermal necrolysis. **(A)** Duck erythema on the back of chemotherapy-induced toxic epidermal necrolysis. **(B)** Toxic epidermal necorlysis after one week of hospitalization.

We also used Zoledronic Acid for Inje(4mg) to relieve the bone pain and destruction before the VRD region treatment. After discovering the patient’s rash (**Figure 2A**), we initially considered it as drug-related rash, allergies, and other diseases as potential causes. To exclude allergies and connective tissue diseases, we conducted tests for allergen and antibody spectrum. However, the results demonstrated allergen negative. As the rash continued to progress, we gradually eliminated other commonly used drugs, and focused on bortezomib or lenalidomide as the most probable culprits. In details, the rash gradually diffused within 3 days, mainly concentrated in the chest, abdomen, back, and scattered on the limbs and face. The area of rash totally over 30% of the whole-body skin. The rash gradually merges, with loose and easily broken epidermis, accompanied by itching and exudation in first week. After the patient initiated lenalidomide treatment, he reported the occurrence of a minor rash, which subsequently resolved upon discontinuation of the medication. By considering the patient’s symptoms in conjunction with a comprehensive review of pertinent literature (20, 21). We postulated that lenalidomide could be attributed as the causative agent for this adverse event. Consequently, we implemented a treatment plan involving the administration of methylprednisolone at a dosage of 60 mg every 12 hours, accompanied by intravenous immunoglobulin infusion at a rate of 0.4 g/kg over a span of five days.

We provided supportive treatment with ebastine to relieve the itching in addition to antibiotic prophylaxis, constant use of a laminar airflow bed, and regular dressing changes. We tested the blood and skin exudate culture twice within one week, and no pathogenic bacterial infection was detected. We also excluded the infection of virus by testing certain antigen and DNA. The patient’s skin and mucosa healed gradually after 10 days of treatment without any residual scarring, and his condition improved significantly. He was discharged within a month of receiving the final treatment with bortezomib and dexamethasone. We administered bortezomib fourth times after the patient recovered from TEN and the patient got no rash again, which confirmed that the rash was caused by lenalidomide. After treatment, the rash was cured, blood pattern was improved, IgG was decreased significantly (PR), albumin was normal, and bone pain was basically relieved.

Subsequent to the resolution of the rash, we conducted lymphocyte subset and cytokine tests to assess the immune response, revealing an elevation in the proportion and quantity of activated T cells, as well as a significant increase in cytokine levels compared to the initial admission. Prior to the onset of Toxic Epidermal Necrolysis (TEN), the levels of IL-6, IL-10, INF-γ, IL-17, IL-4, and TNF-α were within normal ranges. However, at the initiation of the disease, IL-6 levels were measured at 28.8 pg/ml and INF-γ levels at 458.87 pg/ml, subsequently decreasing to IL-6: 28.62 pg/ml and INF-γ: 112.91 pg/ml as the skin condition gradually improved. This declining trend in cytokine levels corresponds to the amelioration of the patient’s condition.

Furthermore, we observed that the proportion and count of natural killer (NK) cells and killer T cells within the lymphocyte subsets were within normal ranges prior to the onset of TEN. However, during the disease onset, the proportion of CD3^+^CD8^+^ cytotoxic T cells significantly increased by 48.9%, while the proportion of CD3+HLA-DR+ late activated T cells exhibited a prominent rise of 39.2%. Additionally, the proportion of NK cells was measured at 17.7%, whereas the proportion of helper T cells exhibited a decline of 28.1%. Consequently, the total count and proportion of T cells demonstrated an increase of 78.2%. Collectively, these findings, in conjunction with the available literature, support the notion of cell activation and an inflammatory overreaction associated with the observed condition.

The standard treatment for TEN usually involves the administration of systemic corticosteroids such as methylprednisolone and intravenous immunoglobulin. However, in this patient’s case, the treatment approach went beyond this and included discontinuing the causative medication (lenalidomide) as well as providing comprehensive supportive therapy. This included the application of ebastine to relieve pruritus, antibiotic prophylaxis, continuous use of a laminar bed, and regular dressing changes. This combined approach was successful in managing the patient’s symptoms and promoting the healing of the skin and mucous membranes without any residual scarring. After completing the final treatment with bortezomib and dexamethasone, the patient was discharged within a month (**Figure 2B**).

The patient experienced pruritus after the first day of oral lenalidomide, ruling out the possibility of long-term use of the drug and bortezomib as causative factors. Notably, there are no previous reports of TEN associated with bortezomib in the literature. Lenalidomide, an immunomodulator commonly used in MM treatment can cause severe reactions but MM patients who developed TEN after using lenalidomide is still rare (22). This patient’s skin was affected in more than 50% of the body surface area, with a severity of illness score for TEN (SCORTEN) of 3, indicating a 35.3% probability of mortality. In **Table 1**, we summarized the data which revealed several previous cases about lenalidomide-induced TEN/SJS in MM patients. In PubMed there are 8 case reports about lenalidomide (VRD region or single drug) induced the TEN/SJS with multiple myeloma. 4 cases are Steven-Johnson syndrome induced by lenalidomide. TEN has been reported rarely as adverse event to lenalidomide. We also found 3 cases SJS/TEN induced by lenalidomide:PMF(Del 5q) (28) ,pRCL (29),ATLL (30). We found different does used by these cases, these also taken place the SJS/TEN in clinic. We consider this adverse event doesn’t matter with dose, it happened maybe relevant with certain characteristics of lenalidomide.

**Table 1:** Published cases of clinical features of patients with SJS/TEN induced by lenalidomide.

Sex	Age	Diagnosis	Sore of TEN	dose	Limited or diffused	Treatment	Healed time	Reference
F	73	SJS, MM	≥2	10 mg	diffused	Corticosteroids antihistamine	9 days syndrome improved	Boruah, P. K, et al. (23)
F	69	SJS, MM IgG κ	1	10 mg	diffused	Corticosteroids antiseptics diphenhydramine hydrochloride antibiotics	30 days	Allegra, A, et al. (24)
M	61	SJS, DIC MM IgG λ	≥2	25 mg(first) /10mg(second)	Diffused(first)/limited chest(second)	rh-TM Corticosteroids antibiotics	14 days	Inoue, Y (25).
F	69	SJS, MM IgG κ	≥2	10 mg	Limited (on the leg, 5%of body surface area)	Corticosteroids topical antiseptics	30 days	Musolino, C et al. (26)
M	61	TEN MM IgA κ	≥2	25mg	Diffused (10-30% of body surface area)	Corticosteroids supportive treatment	21 days	Wäsch, R, et al. (27)
M	69	TEN, MM	3	25mg	Diffused (more than 30% of body surface area)	IVIG Corticosteroids antibiotics supportive treatment	30 days	Our case

Lenalidomide is a widely accepted immunomodulatory agent that enhances antitumor immune responses by activating Th1 cells, leading to increased cytokine secretion and clonal T cell proliferation, followed by enhanced cytotoxic CD8+ T cell function and NK cell activation (31, 32). However, these effects, which can increase inflammation, are also associated with the pathogenesis of SJS/TEN, as demonstrated in this case report. Thalidomide, another immunomodulatory drug, is less effective in stimulating T cells and NK cells than lenalidomide, but it has also been associated with SJS/TEN, especially in elderly male Asian patients with hematological malignancies (33, 34).

Bortezomib is a potent and selective inhibitor of the proteasome complex, widely used as the standard of care for treating multiple myeloma. However, its usage can result in severe side effects such as Stevens-Johnson Syndrome (SJS), as reported in a 71-year-old female patient undergoing chemotherapy. Additionally, there have been rare reports of TEN when combined with bortezomib, and no established protocols for overcoming these negative effects. In this case report, we present a male patient who suffered from TEN after receiving the VRD regimen. We describe our approach to managing these side effects, including the use of antibiotic prophylaxis, continuous use of a laminar bed, and other supportive measures. Through these interventions, we were able to help the patient recover from TEN and strengthen the therapeutic effects of chemotherapy.

## 3 Conclusion

The VRD regimen has been a standard treatment for MM patients for several years and has shown satisfactory therapeutic outcomes. The overall response rate(partial response or better) in MM patients receiving VRD therapy is over 80% (20). The median survival rate in the treatment group has also doubled. However, side effects are inevitable, including anemia, thrombocytopenia, lymphopenia, and neutropenia.

In this case report, we describe a patient who developed SJS/TEN following VRD treatment for multiple myeloma. However, after hospitalization and supportive treatment, the patient was able to recover with a highly efficient immune response.

It is noteworthy that drug-induced SJS/TEN is more common in Asians than Europeans, accounting for 70%–80% of cases (35–37). This may be due to differences in human leukocyte antigen (HLA) alleles between Asian and European populations (38). And the most commonly used systemic treatment for SJS/TEN is glucocorticoid therapy, administered following strict guidelines. However, prolonged use of glucocorticoids can suppress immune function and increase the risk of infection and multi-system dysfunction. Studies have failed to demonstrate reduced mortality with this treatment approach. Retrospective studies have shown promising clinical benefits with a gradual reduction of corticosteroid dosage (1.5~2 mg/kg per day prednisone or equivalent methylprednisolone) (39). The use of systemic glucocorticoids alone for SJS/TEN is controversial as they can inhibit the cytotoxic effects of T cells and NK cells (40). Thus, balancing the immune response by inhibiting immune toxicity to prevent SJS/TEN while inducing a cytotoxic immune response to maintain T cell function to kill tumor cells is a controversial issue.

Lenalidomide, as an immunomodulatory agent, enhances the antitumor immune response mediated by Th1 cells, leading to increased cytokine secretion and clonal T cell proliferation (21, 41). As a result, the cytotoxic CD8+ T cell function is enhanced, and natural killer (NK) cells are activated. However, these effects may also contribute to the pathogenesis of SJS/TEN (21, 41).

**Figure 3:**
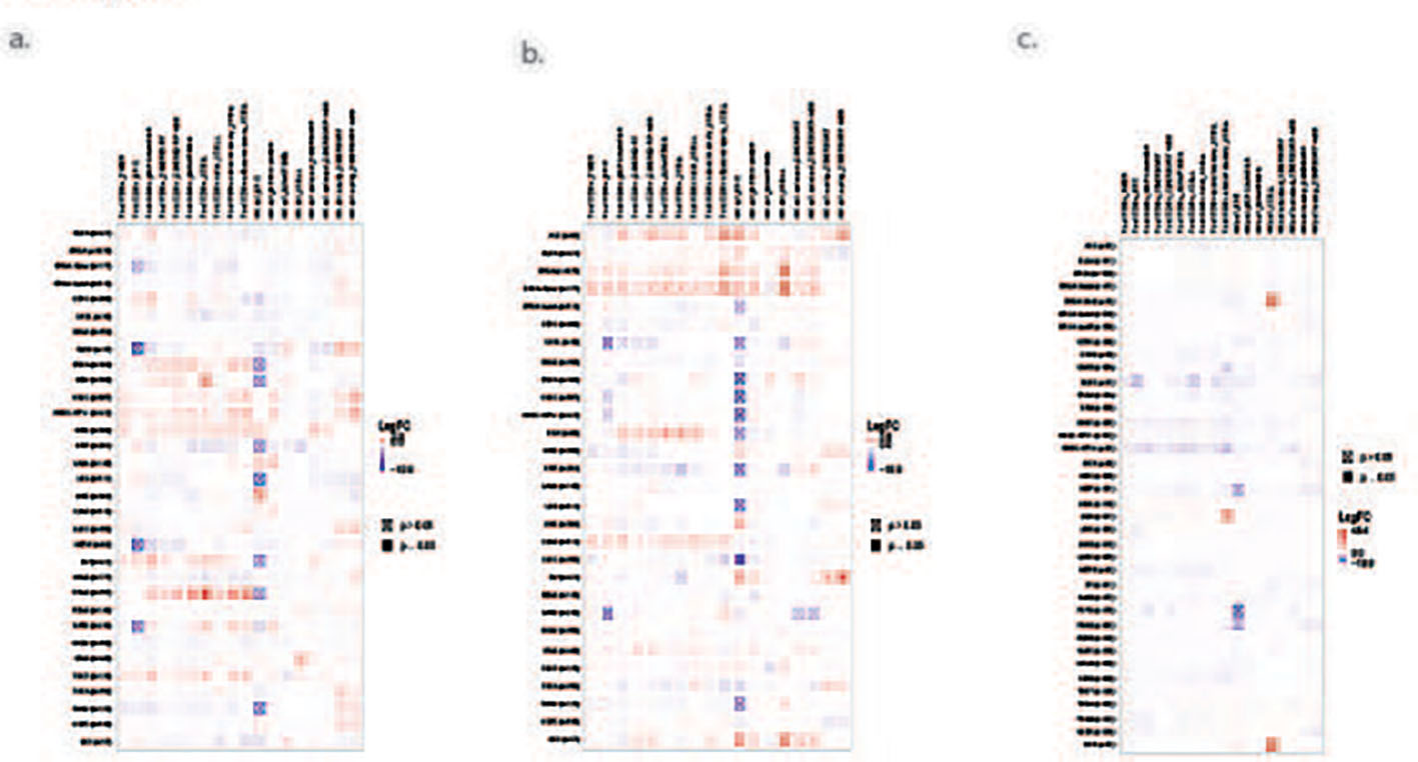
Mutated genes in MM stimulate NK cells and T cells in various other cancer types. **(A)** KRAS; **(B)** NRAS; **(C)** TP53.

In this case report, we carefully monitored the patient’s symptoms and promptly identified the skin-related issues. This enabled us to discontinue chemotherapy and document the progression of the patient’s illness. The potentially life-threatening effects and high mortality rate associated with SJS/TEN make this case report a valuable reminder to both patients and physicians of the risks involved. It is worth noting that the variation in HLA between Asians and Europeans has prompted clinical trials to explore the effectiveness of HLA-B*1502 in preventing SJS/TEN (42). Moreover, the analysis of pre-clinical and clinical data published on Timer2.0 suggests that the frequently mutated genes (such as KRAS, NRAS, and TP53) observed in multiple myeloma may also activate NK cells and CD8+ T cells in other cancer types, indicating their potential to induce an immune response across different malignancies. (**Figure 3**) (43, 44). By increasing awareness of these findings not only in MM but also in a wide range of cancers, we aim to prevent future occurrences of SJS/TEN and enhance patient outcomes.

## Data availability statement

The original contributions presented in the study are included in the article/supplementary material. Further inquiries can be directed to the corresponding author.

## Ethics statement

The studies involving human participants were reviewed and approved by Dongyang People’s Hospital Ethics Committee. The patients/participants provided their written informed consent to participate in this study. Written informed consent was obtained from the individual(s) for the publication of any potentially identifiable images or data included in this article.

## Author contributions

RX and GW collected the clinical data and RX, GW and MW contributed to the manuscript's writing. LZ provided valuable insights and advice, particularly in the discussion section. XW, WC, and CW assisted in managing the patient and provided guidance and photographic evidence. Finally, MW reviewed and edited the manuscript. All authors have thoroughly read and approved the final version of the manuscript.
